# Myeloid sarcoma of the heart—A clinicopathological correlation

**DOI:** 10.1007/s00508-024-02478-3

**Published:** 2024-12-06

**Authors:** Kristijan Skok, Martin Zacharias, Nicolas Verheyen, Stefan Hatzl, Laura Scholz, Cord Langner, Gerald Hoefler, Fotini Rosi Vagena

**Affiliations:** 1 Diagnostic and Research Institute of Pathology, Medical University of Graz, Stiftingtalstraße 6, 8010 Graz, Austria; 2 Division of Cardiology, Department of Internal Medicine, Medical University of Graz, Medical University of Graz, Auenbruggerplatz 15, 8036 Graz, Austria; 3 Division of Intensive Care Medicine, Department of Internal Medicine,, Medical University of Graz, Auenbruggerplatz 15, 8010 Graz, Austria

**Keywords:** Cardiomyopathy, Acute myeloid leukemia, TP53, Rare diseases, Clinical pathology conference

## Abstract

A 63-year-old woman with a history of acute myeloid leukemia followed by stem cell transplantation presented with acute heart failure. Transthoracic echocardiography revealed a preserved left ventricular ejection fraction with severe ventricular hypertrophy and signs of elevated filling pressures indicating infiltrative cardiomyopathy. She died from cardiac arrest due to cardiogenic shock. The autopsy revealed an enlarged heart with a fish-flesh appearance. Here, we describe a rare case of a myeloid sarcoma of the heart.

## Introduction

A 63-year-old female with a background of acute myeloid leukemia followed by stem cell transplantation presented with acute heart failure. Transthoracic echocardiography revealed thickening of left ventricular walls and decreased systolic function. She died of decompensated heart failure due to cardiac myeloid sarcoma, with an autopsy revealing an enlarged heart with a fish-flesh appearance.

## Case report

### Clinical presentation

The patient was hospitalized for congestive heart failure with bilateral pleural effusions and elevated C‑reactive protein levels. She had a history of acute myeloid leukemia (AML), with the last positive biopsy at the end of 2022, confirmed by PCR analysis showing a TP53 mutation. Following the initial diagnosis, she underwent treatment with a liposomal formulation of cytarabine and daunorubicin (CPX-351) for 1 month. The biopsy at that time revealed a TP53 mutational status with variant allele frequencies (VAF) of 65.13%. Subsequently, she received a course of darubicin, fludarabine, cytarabine, granulocyte colony stimulating factor (G-CSF, Ida-FLAG protocol) combined with venetoclax; however, follow-up biopsy results continued to show a positive TP53 mutational status. Another treatment regimen with decitabine and venetoclax was administered but the subsequent biopsy again revealed persistent disease, although the VAF had decreased to 33.70%. One month later, the patient underwent stem cell transplantation. A biopsy following the transplantation showed a significantly reduced disease burden, with the TP53 mutation VAF dropping to 0.08%. Chimerism analysis from bone marrow aspirates and peripheral blood using the AmpFlSTR®Identifiler Plus PCR Amplification Kit (Thermo Fisher Scientific Inc., Vienna, Austria) showed 100% donor cells. Repetitive TP53 mutation analysis in bone marrow aspirates (the last one performed 2 months before death) showed only minimal molecular residual disease with VAF ranging from 0.00% to 0.16%. Follow-up routine bone marrow biopsies (the last one performed 2 months before death) were morphologically and immunohistochemically negative for AML.

**Fig. 1:**
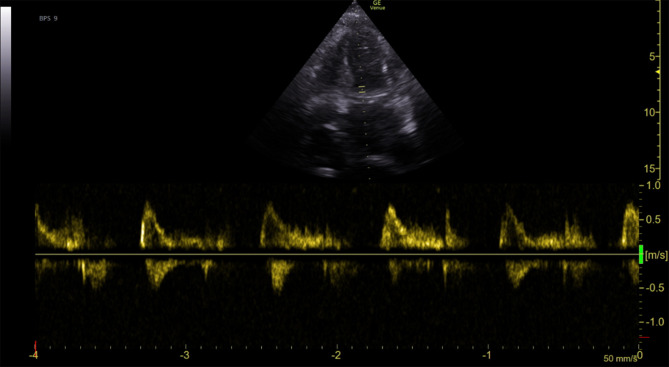
Transthoracic echocardiography showing a hypertrophic left ventricle with hyperdynamic systolic function and restrictive filling pattern

Nevertheless, the patientʼs overall health started to deteriorate. Despite diuretic and antibiotic treatment, the clinical condition gradually worsened and she complained about vertigo and dyspnea. Arterial blood gas analysis performed 2 weeks after admission due to hyperventilation indicated severe lactic acidosis (lactate 16 mmol/L, pH 7.2). At admission to the cardiac care unit (CCU), transthoracic echocardiography showed a hypertrophic left ventricle with hyperdynamic systolic function and restrictive filling pattern (Fig. 1) compatible with the diagnosis of heart failure with preserved ejection fraction due to infiltrative cardiomyopathy. Despite all measures taken lactic acidosis escalated further and 6 h after CCU admission the patient had a cardiac arrest in the context of pulseless electrical activity and passed away.

An autopsy was requested and performed. The questions at autopsy, posed by the clinical colleagues were: 1) cause of death and 2) signs of an infiltrative cardiomyopathy (e.g., amyloidosis)?

### Important autopsy findings

**Fig. 2:**
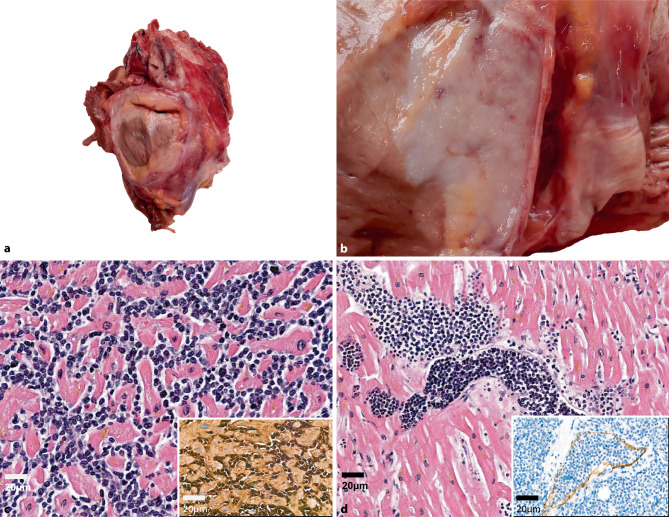
**a** Macroscopic appearance of the heart during autopsy and **b** in close up, showing a fish-flesh-like appearance, **c** Infiltration of blasts into the myocardium (HE, 40 ×), insert of **c** Immunohistochemical stain for CD13 (40 ×). **d** Infiltration of blasts into lymphatic vessels (HE, 20 ×), insert of **d** Immunohistochemical stain for D2-40 (40 ×)

During autopsy, the organs, except the heart, were macroscopically within reference values and without apparent signs of malignancy. The heart was enlarged and weighed 930 g (normal 280–340 g in males and 230–280 g in females) (Fig. 2a, b). The cut surface (anterior, posterior walls and septum) displayed a smooth, homogeneous, pink-tan colored “fish-flesh” appearance. The coronary arteries showed mild sclerosis and the heart walls had signs of severe hypertrophy. The trabeculae and papillary muscles of the left heart were moderately flattened and those of the right heart were flattened as well. For establishing an accurate diagnosis at the time of autopsy a frozen section was performed. On the frozen section, the sample from the heart displayed a diffuse round cell infiltrate that reached from the epicardium to the endocardium. A preliminary diagnosis of malignant round cell neoplasia, most probably recurrence of the primary disease, was formulated and communicated to the clinical colleague.

### Histopathological and molecular findings

Samples from the heart displayed sheets of immature mononuclear cells. On immunohistochemistry, the tumor cells displayed the following phenotype: strongly positive for CD13, CD33, p53 and focal positivity for CD14, negative for CD3 and CD20. The phenotype was concordant with the primary report. Additionally, the cells were seen in lymphovascular spaces, which were identified by D2-40 staining. Samples were also taken from the lungs, kidneys, liver, spleen and bone marrow. Blast cells such as in the myocardium could not be found in any other organs, including bone marrow. This was verified with the use of the same antibody panel as described before. Samples from the heart and bone marrow were also used for mutational analysis. The *TP53* Amplicon-Panel (manufactured in house, not commercially available) found the same pathogenic mutation as was reported in the primary sample: *TP53*: p.C238Y. The sample from the heart had a VAF of 93%, indicating a concomitant allele loss. The same mutation with VAF of 1.26% was detected in the bone marrow, which was seen only after specific visual evaluation. Based on these findings a diagnosis of a recurrence of the AML in the heart was made. Such a constellation is also called myeloid sarcoma (MS) of the heart.

### Discussion

This case demonstrates a MS of the heart with negative bone marrow biopsy presenting with acute heart failure. The clinical course as well as imaging led the colleagues to believe that the patient might suffer from an infiltrative cardiomyopathy.

According to the WHO (WHO classification of tumours series, 5th ed.; vol. 11), an MS is a tumor mass involving any anatomical site other than bone marrow (i.e., extramedullary) that effaces tissue architecture and is composed of myeloid blasts, with or without maturation [1]. The WHO does not recommend terminology such as chloroma, granulocytic or monocytic sarcoma, or extramedullary myeloid tumor. The localization of the tumor can be at any site of the body. The most frequent sites are skin, soft tissue, lymph nodes, gastrointestinal tract, bones and the head and neck region [1, 2].

The literature shows that primary cardiac infiltration by myeloid sarcoma is rare, data from autopsies suggest an incidence of cardiac involvement between 8.7% and 20% [3, 4]. Authors who reviewed all reports of acute myeloid leukemia over a period of 12 years found primary myeloid sarcoma to account for only 1.4% of all cases [5]. A search in PubMed with the MESH terms sarcoma, myeloid and heart, showed only 19 results (from 2003 to 2024 all available results). Based on these results, this entity is not common. The scarcity of data is the reason for the lack of exact guidelines for diagnosis and management. Hematologists are treating this entity mostly based on their clinical acumen [4].

Regarding the question of amyloidosis and hematologic diseases, immunoglobulin-related amyloidosis should be considered in any patient with a multisystemic disease and a monoclonal protein in blood or urine [1, 6]. It can often present as heart failure with preserved ejection fraction, whilst giving the appearance of hypertrophic cardiomyopathy [6, 7]. From a clinical perspective, amyloidosis should be considered as a differential diagnosis in patients with nondiabetic nephrotic proteinuria, heart failure with preserved ejection fraction, nondiabetic neuropathy or unexplained gastrointestinal symptoms and hepatomegaly [7].

## Conclusion

This case highlights the importance of the clinical correlation and the importance of the autopsy in medicine, which has diminished during the years [8] but found renewed interest during the times of the COVID-19 pandemic [9] and, hopefully, will continue to be an avenue for further scientific research [10].
